# A simple and quick sensitivity analysis method for methane isotopologues detection with GOSAT-TANSO-FTS

**DOI:** 10.14324/111.444/ucloe.000013

**Published:** 2021-02-10

**Authors:** Edward Malina, Jan-Peter Muller, David Walton

**Affiliations:** 1 Formerly at Imaging Group, Mullard Space Science Laboratory, Department of Space and Climate Physics, University College London, Holmbury St. Mary, Dorking, Surrey, RH5 6NT, UK

**Keywords:** methane, radiative transfer, GOSAT, isotopologue, SWIR, education, outreach

## Abstract

Measurements of methane isotopologues can differentiate between different source types, be they biogenic (e.g. marsh lands) or abiogenic (e.g. industry). Global measurements of these isotopologues would greatly benefit the current disconnect between ‘top-down’ (knowledge from chemistry transport models and satellite measurements) and ‘bottom-up’ (*in situ* measurement inventories) methane measurements. However, current measurements of these isotopologues are limited to a small number of *in situ* studies and airborne studies. In this paper we investigate the potential for detecting the second most common isotopologue of methane (^13^CH_4_) from space using the Japanese Greenhouse Gases Observing Satellite applying a quick and simple residual radiance analysis technique. The method allows for a rapid analysis of spectral regions, and can be used to teach university students or advanced school students about radiative transfer analysis. Using this method we find limited sensitivity to ^13^CH_4_, with detections limited to total column methane enhancements of >6%, assuming a desert surface albedo of >0.3.

## Statement of robustness

The potential impact of methane and other greenhouse gases (GHGs) on the global environment is recognised at the highest levels of government, as was shown in the recent signing of the COP21 treaty in Paris. Atmospheric methane is composed of differing isotopic concentrations, with ^12^CH_4_ and ^13^CH_4_ representing ~99% of the total methane concentration. Previous studies have shown that the ratio of these two main ‘isotopologues’ can indicate if the measurement is from a biological or non-biological source. Therefore, the exploitation of this known ratio using new measurement techniques on current GHG measuring satellites is timely as well as necessary; potentially allowing for source apportionment on a global scale. This paper demonstrates a unique assessment towards determining the feasibility of retrieving the main methane isotopologues concentrations in the Earth’s atmosphere, using the nadir-sounding instrument the Greenhouse Gases Observing Satellite–Thermal and Near Infrared Sensor for Carbon Observations–Fourier Transform Spectrometer (GOSAT-TANSO-FTS). The methods used in this paper are designed so that advanced school students or early university students can easily apply them, which is important in the context of science outreach and citizen engagement.

## Introduction

### Global context

The impact of methane on the environment and its potential for global warming is well documented [1]. Wuebbles and Hayhoe [2] state that the increasing levels of methane in the atmosphere significantly affects the levels of ozone, water vapour (in the stratosphere), hydroxyl radicals and numerous other atmospheric compounds which result from the oxidation of methane [3]. All these occurrences lead to detrimental effects on the chemistry of the atmosphere (e.g. the formation of tropospheric ozone, and the depletion of atmospheric methane sinks), as well as the absorption of infrared (IR) radiation causing atmospheric heating [3]. The total global methane budget is not currently well understood, as is exemplified by multiple contrasting theories for the stall of the global methane concentration between 2000 and 2006 after a century of increase, and then a subsequent rise from 2014 [4]. Aydin et al. [5] suggest that the drop in global methane output is due to a reduction in the fossil fuel sources of methane, through observations of global concentrations of ethane, which can be used as a global indicator of anthropogenic methane. However, in a completely contrasting view, Kai et al. [6] assert that the reduction in global methane output is in fact due to a reduction in microbial methane from the Northern Hemisphere; while other authors [7–9] suggest that fluctuating hydroxyl radical concentrations are a potential cause of global methane variations. It is therefore important to understand how and where methane is released, and to develop more sophisticated methods of methane detection that will allow for greater understanding of the processes behind methane generation, and how they will affect the global environment.

Methane gas may be formed through multiple natural and anthropogenic processes, including microorganism decomposition of cellulose in sediments under reducing conditions, the breakdown of gas hydrates including clathrates, and thawing permafrost in arctic and subarctic conditions. Melting of the permafrost is a topic of particular concern, with the Arctic warming faster than any other part of the Earth. The Arctic, currently a minor source of methane, could become a major source over the coming century due to warming [10]. Methane emissions from the Arctic are a particularly complex issue, with up to 33% of the world’s organic carbon stored within the Arctic permafrost [11], and vast reserves of methane being stored in crystalline clathrate structures [12]. Yet there is no consensus on how and when these carbon reserves will enter the atmosphere; new data and methods are required to address these uncertainties. Other important processes include; geological processes in the Earth’s crust reaching the surface through features such as mud volcanoes or soil exhalation, catagenesis, metamorphism of coal and dispersed organic matter, as well as during petroleum maturation. Anthropogenic sources such as industry by-products (e.g. leaks from gas plants) and agriculture (e.g. livestock or rice paddy fields) must also be considered as being highly significant [3, 13]. Industrial by-products imply that fossil fuels can be detected by the type of methane gas given off by their formation and exploitation [14, 15]. Towards this end many satellite missions have been focused on trying to measure fossil fuel sources using their methane emissions, including the Japanese Greenhouse Gases Observing Satellite (GOSAT) [16, 17], which was designed specifically for this purpose.

Atmospheric methane consists of a number of different isotopologues (molecules that vary according to their isotopic composition), the main four being ^12^CH_4_ accounting for roughly 98% of atmospheric methane, ^13^CH_4_ making up roughly 1.1% of atmospheric methane and CH_3_D, present in very small concentrations (roughly 0.06%), with all the other isotopologues present in tiny amounts. The ability to distinguish spectroscopically between the isotopologues of methane can potentially allow the determination of the nature of the source of methane emissions (either biogenic, thermogenic or abiogenic), by taking the ratio of the concentration of ^12^CH_4_ and ^13^CH_4_ isotopologues [4, 18, 19]. This method has been previously used effectively for *in situ* terrestrial studies and it is this relationship that is the focus of this study. Currently there are limited global measurements of separated methane isotopologues, the majority of measurement sites falling under the National Oceanic and Atmospheric Administration (NOAA) (www.esrl.noaa.gov/gmd/ccgg/trends_ch4/) as well as a small number of other independent organisations [4]. Based on this limited spread of measurement sites, the existence of a satellite instrument that can differentiate between methane isotopologues would expand the global knowledge of methane distributions. It has been achieved in the upper troposphere and lower stratosphere with solar occultation limb viewing instruments [20, 21], and is hoped to be achieved with dedicated potential future instruments [22].

### GOSAT and measuring radiance

The GOSAT-TANSO-FTS measures solar backscatter radiance, such that solar irradiance passes through the atmosphere, is reflected off the surface of the Earth, and passes back through the atmosphere where GOSAT-TANSO-FTS measures the radiance (i.e. light magnitude). As this light passes through the atmosphere, it is absorbed at specific frequencies determined by the gases the light passes through. Absorbing this light causing the atoms of the specific gas to change energy levels, these jumps are characterised by spectral lines of finite width. Knowledge of the position of spectral lines for methane (or other gases), means it is possible to calculate how much energy was absorbed by these gases, and therefore how much of this gas is in the path the light travelled through the atmosphere [23].

The radiance received at the instrument due to absorption in the Sun–Earth–GOSAT light path is determined by the Beer–Lambert law [24], expressed by the equation

(1) $$\text{I}(\lambda )={\text{I}}_{0}(\lambda )e^{-\sigma \text{C}(\lambda )\text{x}},$$

where I refers to the intensity of the incident radiation at wavelength λ, given an optical path of thickness x, and I_0_ is the intensity of the initial incident light or radiation, C is the density of the light path (or concentration of molecules) and σ is the absorption cross section (or the likelihood of absorption by a given molecule).

**Figure 1:**
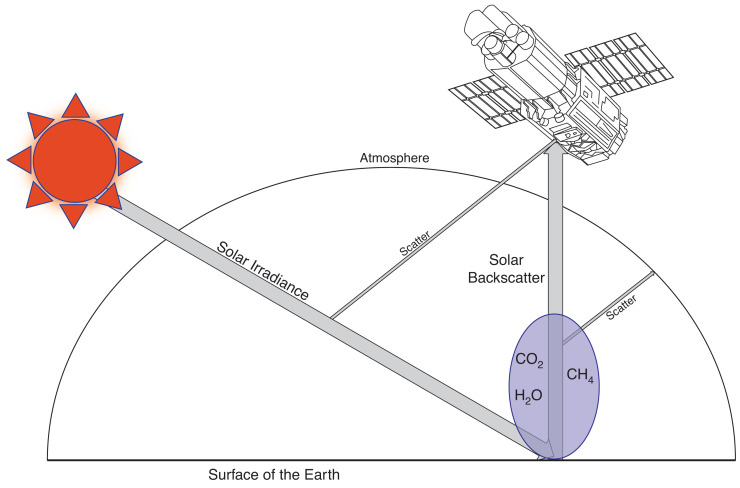
Schematic representation of GOSAT measuring solar backscatter light.

A representation of the standard operations of GOSAT is identified in Fig. 1, where I(λ) is what is received at GOSAT-TANSO-FTS, and σ in the case of this paper refers to methane spectral lines. However, Equation 1 is not directly applicable to what GOSAT-TANSO-FTS measures, as Equation 1 assumes a constant density across the light path. The density of the atmosphere (C) is not constant, meaning Equation 1 must be separately applied for multiple atmospheric layers, in order to accurately measure absorption over a long distance. Larger particles in the atmosphere (e.g. aerosols) can scatter radiation away from the main light path, meaning the difference between I and I_0_ is not purely due to absorption. These absorption and scattering properties vary depending on what region of the electromagnetic spectrum is being observed. Meaning that prior to attempting to measure the concentration of trace gases in the atmosphere, the most optimum portion of the electromagnetic spectrum must be identified.

The aim of this paper is to identify spectral regions where the main methane isotopologues (^12^CH_4_ and ^13^CH_4_) can be detected with the existing GOSAT-TANSO-FTS. Such studies are typically performed using the information content (IC) analysis method described in [25], examples of which are also reported in [26–28]. IC analysis is a powerful tool, but has several significant challenges associated with its use. Firstly, on its own the IC analysis cannot be used to estimate atmospheric trace gas concentration as it is an analysis method and not a full retrieval algorithm (such as in [28–30]). Secondly, there is a substantial step in effort required to convert the IC analysis method into a retrieval tool capable of trace gas estimation (in terms of computation, analysis methods, etc.). The current algorithms used to produce trace gas concentrations from instruments such as GOSAT-TANSO-FTS or the recently launched Sentinel-5P/Tropospheric Monitoring Instrument (TROPOMI) are the results of multi-year efforts, built on experience with older instruments [e.g. the SCanning Imaging Absorption SpectroMeter for Atmospheric CHartographY (SCIAMACHY) or similar]. Therefore, new research into satellite trace gas retrieval must rely on one of these well-established algorithms, or embark on an expensive development programme.

In this paper we propose using a simple residual radiance analysis technique to identify the suitability of GOSAT-TANSO-FTS for detecting ^13^CH_4_, and the ratio of ^13^CH_4_ and ^12^CH_4_ known as δ^13^C, which is based on the IC analysis method. Although the residual radiance analysis technique is not as sophisticated as the optimal estimation method (OEM) of Rodgers [25], it remains relevant in the context of trace gas detection/retrieval for its ease of use, and quick applicability. Fundamentally, the residual radiance technique is an excellent starting point for becoming familiar with the OEM, and could be an important aspect for advanced school students or university students.

The paper is structured as follows:

- Abstract, Statement of Robustness and Introduction (three sections);
- Description of tools and methods used in study;
- Outline of the results;
- Discussion of methods and results;
- Conclusion.

## Experimental design and starting assumptions

### Methane source isotopologue composition

The isotopic composition of atmospheric background methane and methane sources has been studied at some length [4, 8, 31, 32], especially the four key isotopologues ^12^CH_4_, ^13^CH_4_, ^12^CH_3_D and ^13^CH_3_D. These papers effectively describe how the ratios of methane isotopologues (often referred to as ‘δ’ values) can be used to identify the nature of the source. Normally the metrics δ^13^C and δD are used to define the ratio of isotopologues at the source. The δ^13^C ratio is defined as

(2) δ13C=((13C12C)sample(13C12C)standard−1)×1000000,

where δ^13^C is generated by taking the ratio ^13^C:^12^C of the gas sample under investigation, and dividing it by a base ratio (or standard ratio) taken from the established literature known as the Vienna Pee Dee Belemnite, which then determines how far the sample in question deviates from the standard [33]. A large negative value indicates that the sample is depleted in ^13^C. Large negative values tend to be associated with biogenic sources of methane, while values closer to 0 are largely from industrial sources.

The methane-to-deuterium-based methane ratio is known as δD and is calculated using a similar method to the calculation of δ^13^C, this ratio divided by an established base ratio taken from the established literature is known as the Vienna Mean Standard Ocean Water. However, as stated earlier, deuterium-based methane is very rare in the atmosphere, and we decided early on to focus solely on ^13^CH_4_ as opposed to CH_3_D.

The main reason for the depletion of the heavier isotopologues in biogenic sources is due to the observation that formation of methane by microorganisms tends to discriminate against ^13^C due to kinetic isotope effects (KIEs), accounting for the low δ^13^C values. Different forms of microorganisms will have different rates of KIEs, thus changing the δ^13^C values with respect to the exact source, however, the precise nature of these KIEs is still poorly understood. In addition, specific plants will vary in their ^13^C signature due to differing photosynthetic enzymes, partially accounting for the range in δ^13^C values noted in microbial sources [34, 35].

### Radiative transfer models – SCIATRAN and ORFM

Radiative transfer models (RTMs) are a fundamental aspect of this work, and a key aspect of this study is focused on providing trace gas investigation methods for independent research. It is difficult to perform trace gas research without the use of an RTM. Developing an RTM from scratch for this project fulfils neither of the quick or simple goals, and we therefore decided to use an open source RTM.

In this study we use the SCIATRAN RTM [36], developed by the SCIATRAN working group at the Institute of Environmental Physics and the University of Bremen, available from http://www.iup.uni-bremen.de/sciatran/index.html SCIATRAN is an RTM capable of solving the radiative transfer equation using multiple numerical methods. SCIATRAN can simulate satellite solar backscatter radiative transfer in both clear-sky and aerosol-loaded conditions. SCIATRAN is versatile and can simulate numerous atmospheric effects such as clouds, fluorescence, advanced bidirectional reflectance distribution functions and others for multiple geometry types. For this study, the simulations from SCIATRAN are run at a spectral resolution of 0.01 cm^−1^ and are convolved with a TANSO-FTS type Gaussian instrument line shape function (ILSF) of 0.27 cm^−1^ full width half max [16]. All simulations include multiple scattering effects, where all Mie scattering effects assume spherical particles. SCIATRAN has a significant pedigree with previous instruments such as SCIAMACHY, and has been previously used in studies relating to GOSAT previously [37].

SCIATRAN uses a climatological database derived from a two-dimensional (2D) chemistry transport model (CTM) described in [38]. All gases, temperatures and pressures are provided in the altitude range 1–95 km for 10° latitudinal bins for all months in a given year. The isotopologue profiles in SCIATRAN are identical to the CH_4_ profile included in the simulated atmosphere. The difference in abundance between CH_4_ and ^12/13^CH_4_ is accounted for in the HITRAN2016 database, which scales the isotopologue line strengths by abundance figures provided by [39]. The advantage of this method is that the complexity of adding an additional trace gas profile to the forward model is reduced, the disadvantage is that this scaling assumes that this abundance ratio is true for the whole globe (which is unlikely to be true).

Scattering is considered in SCIATRAN, both through Rayleigh scattering and aerosol-induced Mie scattering. Rayleigh scattering is not considered in this study as it is minor in the shortwave infrared (SWIR). For aerosol-related scattering SCIATRAN draws upon the LOWTRAN database [40], which can simulate multiple different aerosol types for different layers of the atmosphere. In this study we assume the standard SCIATRAN/LOWTRAN settings for aerosol loading in SCIATRAN.

The spectral line database used in this study is HITRAN2016 [41]. HITRAN2016 builds upon the HITRAN2012 database, but includes an increase in the number of assigned ^13^CH_4_ spectral lines, with Brown et al. [42] indicating a significant jump in the number of and accuracy of ^13^CH_4_ (and ^12^CH_4_) spectral lines in comparison to the previous HITRAN iteration (HITRAN 2008; [43]). HITRAN2016 includes data from recent studies such as [44], which contain numerous additional line assignments in the spectral range of GOSAT-TANSO-FTS band 2. However, it is not suggested that there are any updates to the ^13^CH_4_ line lists in band 4 of TANSO-FTS.

In addition to SCIATRAN, we also employ the Oxford Reference Forward Model (ORFM; [45]), developed at the University of Oxford, and available at http://eodg.atm.ox.ac.uk/RFM/ We do not use the ORFM in the residual radiance calculations described in the following sections, but rather to simulate atmospheric transmittance and optical depth. This is because the ORFM allows for quick and easy transmission (and absorption) calculations in all of the wavelengths of interest in this study. ORFM is not used for the residual radiance study as a ‘sun’ is not included in the radiance calculations, and scattering is not included.

### GOSAT-TANSO-FTS

The Japanese Aerospace Exploration Agency (JAXA) launched GOSAT in 2009; GOSAT was the first satellite specifically designed to measure GHG emissions around the globe. The GOSAT project is a joint effort between the Ministry of the Environment (MOE), the National Institute for Environmental Studies (NIES) and JAXA [16, 46]. GOSAT originally had a 6-year lifespan, but this has since been extended. Its replacement GOSAT-2 was launched in October of 2018.

The key instrument on GOSAT is the TANSO-FTS, which measures the radiance of sunlight reflected from the Earth’s surface through the atmosphere in three separate bands: the main band of interest in this study is band 2 which measures radiance in the wavenumber range 5814–6410 cm^−1^ (1.56–1.72 μm), with a sampling interval of 0.2 cm^−1^. GOSAT-TANSO-FTS has a fourth band that measures emissions spectra in the thermal infrared (TIR) between 699 and 1799 cm^−1^ (5.56–14.3 μm) [16, 46].

GOSAT has a history of providing reliable estimates of the global distributions of methane and carbon dioxide [30, 47–49] since its launch. With its high spectral resolution and high signal to noise ratio (SNR), GOSAT was judged to be a good candidate for detecting methane isotopologues, and therefore prompted this investigation. There are other instruments for measuring methane isotopologues from orbit, for example, SCIAMACHY and TROPOMI. SCIAMACHY has a significantly lower spectral resolution (1.5 cm^−1^) and has been found to have poor single sounding precision. Buchwitz et al. [50] state that SCIAMACHY registers a maximum single sounding measurement precision of 30 ppbv, which is unlikely to be sufficient for the retrieval of ^13^CH_4_, where the total column concentration of ^13^CH_4_ is roughly 20 ppbv. The recently launched TROPOMI is a possible candidate for methane isotopologues measurements, TROPOMI contains a push-broom spectrometer and sacrifices spectral resolution (0.45 cm^−1^) for much increased SNR. TROPOMI is likely to be investigated in the future for methane isotopologue detection.

### Study structure and methods

The following subsection discusses the structure of the research study. The key aims are to show the following under realistic atmospheric conditions:

- The optimal regions in bands 2 and 4 of the GOSAT-TANSO-FTS for ^13^CH_4_ detection.
- Measurable changes in ^13^CH_4_ spectral lines over and above the background contaminating gases, and GOSAT-TANSO-FTS instrument noise.
- The effects of background contaminating gases on any measurable changes.

### Spectral region identification

The first step of this study is to make an initial assessment as to where the least contaminated regions for ^13^CH_4_ may be found in the SWIR and TIR. The strongest absorption lines for methane in the SWIR are present within the wavebands at 1.6 μm and 2.3 μm [42]. However, the GOSAT-TANSO-FTS sensitivity to methane is limited to 1.6 μm, in band 2. In the TIR region there is a broadband methane absorption feature at 7.7 μm, which is covered by band 4 of TANSO-FTS. We therefore set-up a simulation scenario with ORFM in order to pick out the maximum absorption points for the ^13^CH_4_, outlined in Table 1.

**Table 1.** The conditions used by ORFM in generating SWIR Absorption from an assumed GOSAT-TANSO-FTS-like instrument. All conditions are taken from MIPAS Model atmospheres [51].

Condition variables	Value
Wavelength range	1600–1700 nm
	7600– 8300 nm
Background gases	H_2_O, CO_2_ and N_2_O at standard model concentrations
Instrument altitude	666 km
Solar zenith angle	30°
Atmospheric model	UoL MIPAS Model
Spectral line database	HITRAN 2016
Spectral resolution	0.01 cm^−1^
Viewing profile	Nadir

ORFM, Oxford Reference Forward Model; UoL, University of Leicester; SWIR, shortwave infrared.

**Figure 2:**
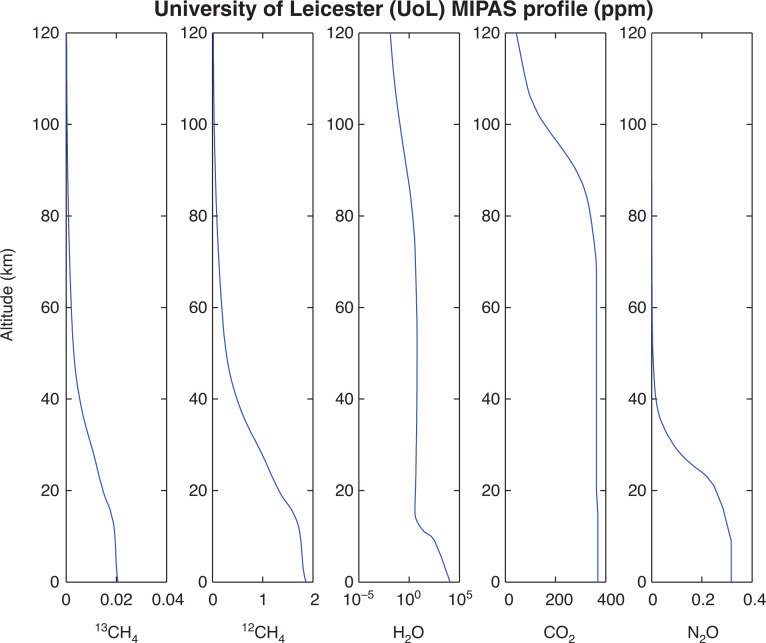
Volume mixing ratio profiles of the main gases of interest CH_4_, H_2_O and CO_2_ in ppm from 0–120 km altitude [51], adapted from [27].

The atmospheric model used in this assessment provides a high number of vertical levels and gas concentrations at more recent magnitudes (2002) than the standard mid-latitude model atmospheres (which were designed in the 1970s), and was originally designed to aid in Michelson’s interferometer for passive atmospheric sounding (MIPAS) retrievals [51]. An example of the atmospheric profiles of three gases from this model is shown in Fig. 2.

GOSAT-TANSO-FTS measures the column average density of methane and carbon dioxide (XCH_4_, XCO_2_); therefore, using the pressure profiles captured in the University of Leicester (UoL) MIPAS profile, the column-averaged densities can be calculated.

The strongest absorption regions of the methane isotopologues are then investigated in order to gain further insight into the influence of contaminant gases on the isotopologues. The ORFM includes the options to simulate absorption as well as radiance, thus giving some insight into the presence of spectral lines of interest. The conditions required to calculate a typical ^13^CH_4_ atmospheric absorption profile are specified in Table 1.

### Detecting changes in the ^13^CH_4_ signal

Background simulated radiance values (containing radiance from the main contaminating gases), are subtracted from the radiances generated from a scenario with elevated concentrations of methane. If this calculated residual difference is greater than the noise radiance known as the noise equivalent delta radiance (NEDL) then it suggests that GOSAT-TANSO-FTS could detect this change in methane concentration. This is known as the residual radiance technique, and has been demonstrated by both Leifer et al. and Roberts et al. [52, 53] as an effective technique for assessing whether changes in concentrations of trace gases can be detected. Roberts et al. [53] state that spectral residuals are often the first step in full atmospheric inversions. Following the method proposed by [53], the residual radiance technique is used to determine the atmospheric conditions when isotopologue retrieval may be possible. The key question to answer is which combination(s) of methane concentration, water vapour concentration and surface reflectance allow for a residual radiance greater than the instrumental noise. This can be determined from the equation

(3) $${\text{F}}_{\text{d}}=|{\text{L}}_{\text{b}}(\text{A},\lambda_{\text{m}})-{\text{L}}_{\text{e}}(\text{A},\lambda_{\text{m}})|-\text{NEDL,}$$

where F_d_ is the detection factor, where any value above 0 suggests that some signal is detectable above the noise limit, and therefore constitutes a detection. L_b_ is the background radiance at the wavelength of the maximum radiance λ_m_ given reflectance A, L_e_ is the atmospheric radiance with elevated methane concentrations (see Table 3) at the wavelength of the maximum radiance λ_m_ given reflectance A and NEDL.

Typically, NEDL can be calculated from knowledge of instrument parameters (dark current etc.), however, these parameters are often kept secret by instrument manufacturers. According to the GOSAT-TANSO-FTS instrument manufacturers at JAXA the GOSAT-TANSO-FTS L1B product [interferograms (L1A data) are converted into radiance spectra via a Fourier transform, including some data screening routines]. They contain two separate elements: real spectra (equivalent to the radiance spectra of interest in trace gas retrieval), and imaginary spectra which are equivalent to noise from FTS theory. The implication of this is that the noise from the spectrum of a particular retrieval can be extracted from the L1B spectra. Therefore, we generate a relationship where the noise profile of GOSAT-TANSO-FTS is estimated given a radiance output from real spectra. The steps for generating this profile are as follows: Extract the real and imaginary spectra from several L1B data GOSAT-TANSO-FTS band 2 products, in order to get variation in radiance output based on the location and surface characteristics of the retrieval. Calculate the root mean square (RMS) of the off-band imaginary spectrum radiance (off-band being the region where the indium gallium arsenide detector is not sensitive to the incident radiation due to an optical band pass filter present in the instrument). This is equivalent to the inherent instrument noise, and the RMS of the on-band (where the detector is sensitive to measured radiance) real spectrum for multiple retrievals. This builds up a profile of how instrument noise varies with received radiance at the detector (dominated by shot noise). The square of the RMS imagery spectrum radiance values is then plotted against the RMS of the real spectrum radiance values; this builds up a profile of how the noise is dependent on the spectral radiance, as well as highlighting what the basic instrument noise is. This allows for a mathematical relationship to be generated, meaning that for any given particular retrieval radiance, a specific noise value can be attributed to it. Using a random selection of 400 GOSAT L1b spectra downloaded from the GOSAT Data Archive Service (https://data2.gosat.nies.go.jp/index_en.html), the relationship was calculated as

(4) $$\text{NEDL}=\sqrt{\left(1.76e^{-8}\text{L}+1.358e^{-11}\right)}\times \text{C},$$

where L is the received radiance (in W/cm^2^/str/cm^−1^) and C is a conversion factor from internal GOSAT units into radiance units. The value of C is available on the GOSAT data archive website in the TANSO-FTS Radiometric Conversion for Band 1–3 document (https://data2.gosat.nies.go.jp/doc/document.html#Document). In this study the NEDL is assumed to be a constant value over the whole spectral range, and we assume that the GOSAT spectra are captured under high gain conditions.

Equation 3 is based on using individual measurements, which will most likely suffer significantly from noise levels. However, as suggested by Roberts et al. [53], the NEDL can be reduced by averaging multiple spectral measurements focusing on the spectral positions with the most ^13^CH_4_ information. In such a case the NEDL reduces with √n, where n is the number of spectral sampling points, described by (modified from [53])

(5) $${\text{F}}_{\text{d}}=\frac{\sum_{\lambda =\text{b}}^{\lambda =\text{a}}\left({\text{L}}_{\text{b}}(\text{A},\lambda_{\text{m}})-{\text{L}}_{\text{e}}(\text{A},\lambda_{\text{m}})\right)}{\text{n}}-\frac{\text{NEDL}}{\sqrt{\text{n}}},$$

where F_d_ is the detection factor over an averaged number of spectral bands, n is the number of spectral bands for combination, between wavelengths a and b. In the normal operation of GOSAT there is no oversampling of measurement points, until the satellite returns to the same orbital path (i.e. only one spectrum is captured per sample point). In this case the method proposed in Equation 5 cannot be used, as repeat measurements are captured under different conditions. However, Kuze et al. [54] describe non-standard operational modes, one of which includes three repeat measurements of the same point for ‘sun glint and limited calibration and validation site observations’. Although not all GOSAT data will be captured in this way, for simulation purposes, it is justified to investigate the effects of averaging three concurrently captured spectra. Indeed, GOSAT has a ‘targeted observations’ mode, where registered researchers can request observations of specific sites, implying that a large number of concurrently captured spectra could be obtained with this method. The exact details of this mode are not published, and are therefore not modelled in this study. Note that the method described in Equation 5 assumes that errors between spectral points are uncorrelated.

The sensitivity of any ^12^CH_4_ and ^13^CH_4_ absorption bands to interfering trace gases and different reflectance conditions must also be considered; the methane absorption windows in the SWIR are typically heavily influenced by water vapour, and therefore any absorption by ^13^CH_4_ is likely to be affected. The influence of water vapour on specific ^13^CH_4_ absorption peaks can be determined from the simple ratio factor (modified from [53])

(6) $${\text{S}}_{\text{f}}=\frac{\frac{\sum_{\lambda =\text{b}}^{\lambda =\text{a}}{\text{L}}_{\text{res}}({\text{W}}_{\text{s}},\text{A})}{n}}{\frac{\sum_{\lambda =\text{b}}^{\lambda =\text{a}}{\text{L}}_{\text{res}}({\text{W}}_{\text{e}},\text{A})}{\text{n}}},$$

where S_f_ is the sensitivity factor, L_res_(W_s_, A) is the residual radiance between background and elevated methane conditions at standard atmospheric conditions between wavelengths, a and b, L_res_(W_e_, A) is the residual radiance between background and elevated methane conditions with elevated water vapour concentrations between the wavelengths, a and b, and n is the number of spectral measurements considered. Note that this method applies to any desired target and interfering species.

It is important to define appropriate atmospheric scenarios in order to determine feasible detection factors, with the key factors being methane concentration in the atmospheric profile and surface reflectance. Numerous total column retrieval methods are based on the ‘scale’ method, where the total column concentration is scaled rather than individual atmospheric layer concentrations modified. Therefore, a range of total column scale factors on which to calculate residuals are specified, appropriate to real world scenarios. The maximum total column XCH_4_ values observed from GOSAT tend to be roughly 1900 ppb [48], equating to a column scaling of 10% (w.r.t. to the MIPAS profile). Very large methane values (>1900 ppb) have been observed by GOSAT in fire affected regions [48], suggesting that although >1900 ppb values are possible, they will be found in unique circumstances.

The second key factor, reflectance, can be determined using the online database created by UCL and Noveltis under contract to the European Space Agency (ESA) called ‘A surface reflectance Database for ESA’s earth observation Missions (ADAM)’ available at http://adam.noveltis.com/ [55]. ADAM predicts that the expected Earth’s surface reflectance values at 1600 nm range from 0.1 for densely vegetated areas, to 0.6 for desert regions (e.g. in the United States or the Sahara).

Based on this range of values, a series of simulation conditions and scenarios were generated as specified in Table 2.

**Table 2.** SCIATRAN Simulation conditions for detection study.

Sensor	Surface/atmosphere	Notes
Solar zenith: 30°	Background conditions	
Altitude: 666 km	Reflectance: 0.1, 0.3–0.6	
	H_2_O: As SCIATRAN CTM (November, latitude 45°)	
	CH_4_: As SCIATRAN CTM (November, latitude 45°)	
	Aerosols: As LOWTRAN SCIATRAN standard settings	Maritime/tropospheric in the boundary layer. Background in the stratosphere
	Elevated conditions	
	Reflectance: 0.1, 0.3, 0.6	Vegetation to desert
	H_2_O: As background ×2	Not necessarily realistic, but indicates sensitivity to water vapour
	CH_4_ scale factor: 1.02, 1.04, 1.06, 1.08, 1.10	XCH_4_ values, minimum 1.78 ppm, maximum 1.94 ppm

### Applying to GOSAT-TANSO-FTS L1B spectra

The final step in this process is to determine whether or not the changes shown in the results from the section Detecting changes in the ^13^CH_4_ signal are observable in real L1B spectra. Towards that end, GOSAT L1B spectra were downloaded from the GOSAT Data Archive Service (https://data2.gosat.nies.go.jp/index_en.html), and compared against synthetic spectra, in order to determine what levels of ^13^CH_4_ variation can be expected over real scenes as opposed to synthetic scenes. Unlike in the section Detecting changes in the ^13^CH_4_ signal, direct comparisons of L1B spectra and synthetic spectra are not quite as simple, as all L1B spectra are captured under a wide range of atmospheric, surface reflectance and instrument geometry conditions. But close conditions are required in order to make any comparisons valid. Therefore, we matched the conditions in real spectra as closely as possibly by: 1) Using solar zenith angles and instrument angles identified in the L1B data. 2) Using the geolocation of data capture to inform as to which UoL MIPAS model atmosphere to use. 3) Identify surface reflectance values by fitting reflectance values in 0.001 steps linearly to the synthetic spectra sections until the RMSE difference between the synthetic spectra and the L1B spectra were at a minimum. 4) Convolved the synthetic spectra with the GOSAT instrument line shape model available on the data archive service, and resampled to a 0.2 cm^−1^ grid using a ‘Matlab’ spline interpolation function. 5) Applied a linear shift to the x axis of the L1B spectra, as the wavenumber axis on TANSO-FTS is variable. The magnitude of the linear shift is defined by wavelength differences between large spectral peaks found in both simulated and measured spectra.

Based on these conditions, direct comparisons between synthetic spectra and L1B spectra were made over known regions of ^13^CH_4_ activity in the SWIR spectrum. Several hundred L1B data points from June in 2016 were used in order to provide a wide range of atmospheric and surface conditions.

## Results

### Absorption assessment

#### SWIR

**Figure 3:**
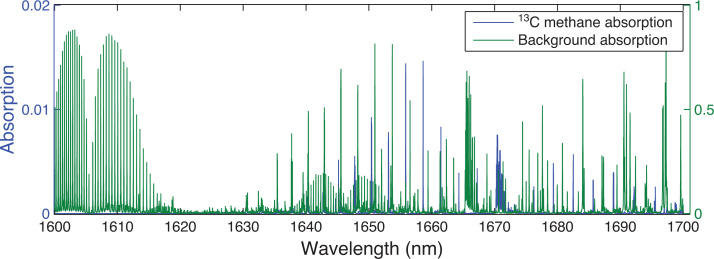
Simulated absorption spectrum from ORFM in the wavelength range 1600–1700 nm, the y scale represents the fraction of radiation absorbed by the molecules under investigation. The blue line represents absorption by ^13^CH_4_ (left-hand scale) and green represents all other key absorbing background gases (CO_2_, H_2_O and ^12^CH_4_) (right-hand scale).

**Figure 4:**
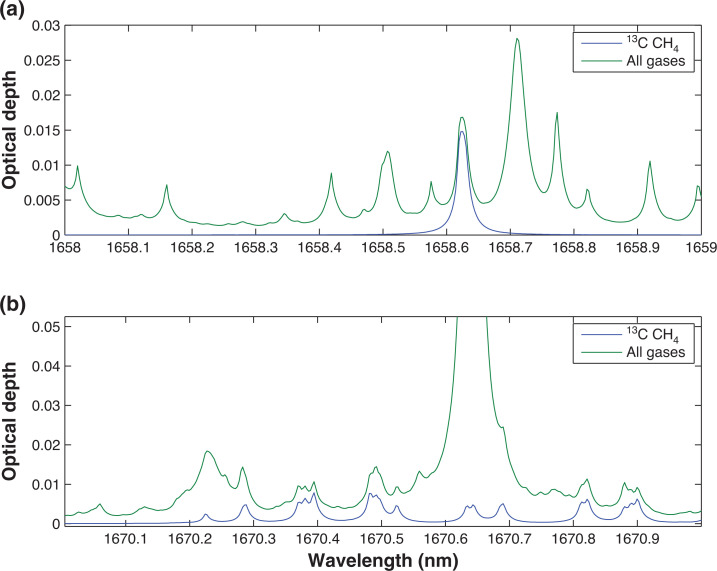
Optical depth covering ^13^CH_4_ absorption points of interest, the green line represents optical depth of all gases present in this portion of the spectrum (CH_4_, CO_2_ and H_2_O), whilst the blue line shows optical depth of purely the methane isotopologue ^13^CH_4_: (a) indicates optical depth in the wavelength range 1658–1659 nm; (b) shows optical depth in the wavelength range 1670–1671 nm. This figure is as figure 5 in [27], but has been updated to reflect the use of HITRAN2016.

Using the atmospheric conditions specified in Table 1, ORFM was used to focus on the 1600–1700 nm region. Figure 3 indicates that it will be challenging to resolve ^13^CH_4_ absorption lines in this spectral region, suggesting that pinpointing ^13^CH_4_ absorption above background gases will be difficult. The strongest/most dense ^13^CH_4_ lines appear to be at 1658–1659 nm and 1670–1671 nm. Focusing on these two spectral regions, the optical depth is explored to determine the effect of background absorbers at these specific wavelengths. Figure 4 makes clear that both of the ^13^CH_4_ spectral regions indicated have similar optical depth values to those of all of the remaining gases, implying that the majority of absorption in these spectral regions is due to ^13^CH_4_. However, the spectral line in the 1658–1659 nm wavelength range clearly shows the least interference from background contaminating gases, therefore suggesting that it is more suited for retrieval. In spite of this, it is obvious that the optical depth of the ^13^CH_4_ lines in this region is very low, and it will therefore be challenging to detect any changes to ^13^CH_4_ in this wavelength range.

#### TIR

**Figure 5:**
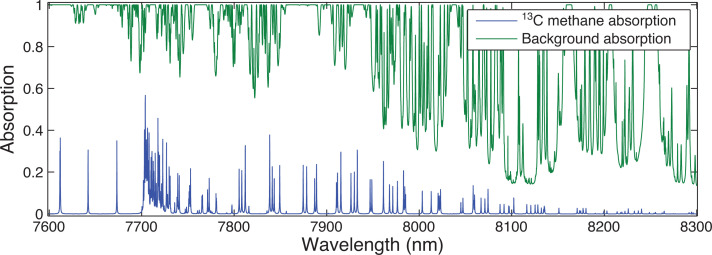
Simulated absorption spectrum from ORFM in the wavelength range 7600–8300 nm, the y scale represents the fraction of radiation absorbed by the molecules under investigation. The blue line represents absorption by ^13^CH_4_ and green represents all other key absorbing background gases (CO_2_, H_2_O, N_2_O and ^12^CH_4_).

Focusing on the TIR band of GOSAT, we perform a repeat analysis of the SWIR. Comparing the strength of ^13^CH_4_ absorption in the TANSO-FTS TIR wavelength range shown in Fig. 5 against that in the SWIR shows a number of striking differences, primarily in the magnitude of the absorption. With the strongest of the ^13^CH_4_ TIR lines having absorption strengths ×40 of their SWIR equivalents. Despite this, background interference is still strong, dominated by water vapour continuum absorption. We now focus on the optical depth of two regions, the 7700–7800 nm region due to the strength of ^13^CH_4_ absorption in this region, and the 8050–8150 nm range due to the lower background absorbance.

**Figure 6:**
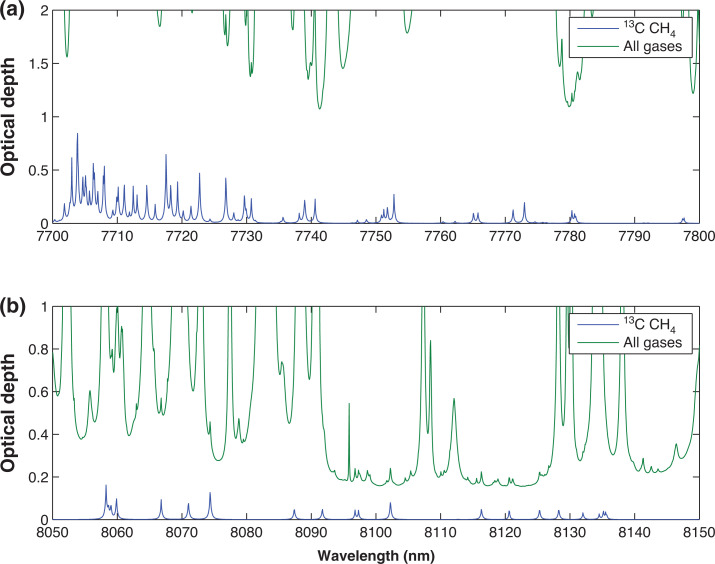
As for Fig. 4, but focused on the wavelength ranges 7700–7800 nm and 8050–8150 nm.

The optical depth survey shown in Fig. 6 demonstrates magnitudes far in excess of the SWIR optical depth in Fig. 4 (especially Fig. 6(a), where the atmosphere is opaque), but as shown in Figs 5 and 6, the background interference on the ^13^CH_4_ signal is significant, with only minor impacts from the ^13^CH_4_ spectral lines. This leaves us with the unenviable position of small optical depth but low background interference in the SWIR, and high optical depth but high levels of interference in the TIR.

Figure 6 suggests that ^12^CH_4_ and other background gases will dominate the residual radiance method for the TIR. Therefore, for this reason, and because measurements in the TIR are often more uncertain than SWIR measurements, exemplified in multiple studies [56–58], we decided to focus on the SWIR in this study. In addition, it has been shown that the SNR on the methane absorption regions in GOSAT are significantly lower than in the SWIR [56, 59], suggesting that the TIR is not ideal for methane retrieval with GOSAT. TIR instruments are heavily based on measuring thermal contrast between atmospheric layers, and because of the lack of such contrast in the lower troposphere, therefore have limited sensitivity near the surface [60, 61]. This suggests that measurements in the SWIR are far more likely to capture methane fractionation at the surface than in the TIR. There are cases with global scenes with high thermal contrast, which will allow for sensitivity to the surface for TIR instruments, however, we believe that the low SNR of TANSO-FTS band 4 is the more important issue, as opposed to surface sensitivity.

### ^13^CH_4_ detectability under standard conditions

**Figure 7:**
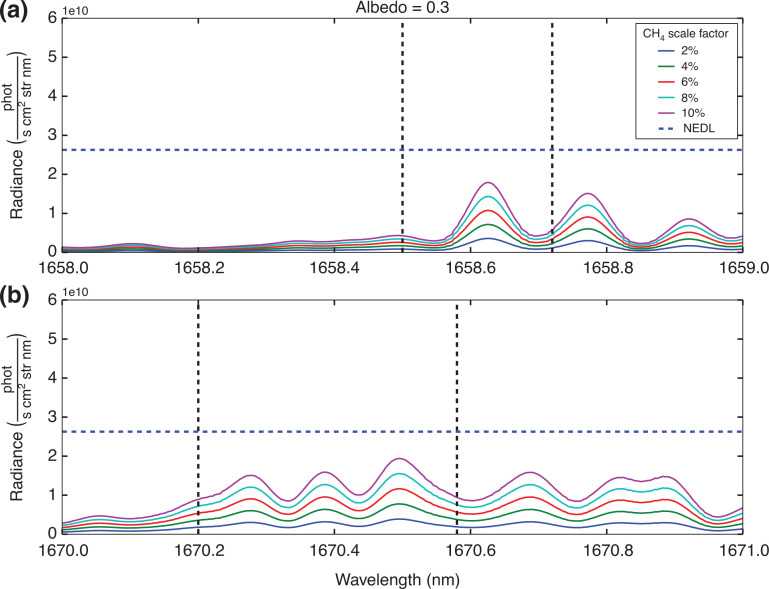
Residual radiance plots based on the simulation conditions highlighted in Table 3, where simulated radiance from the background conditions under the standard ‘day’ scene with a reflectance of 0.3 are subtracted from elevated methane conditions. The residual radiance values are represented by the lines indicated in the legend. The blue dashed line represents the NEDL. The solid vertical dashed lines identify the regions where ^13^CH_4_ spectral lines are prevalent: (a) highlights the ^13^CH_4_ spectral line in the 1658–1659 nm range; (b) focuses on the ^13^CH_4_ spectral line in the 1670–1671 nm range.

**Figure 8:**
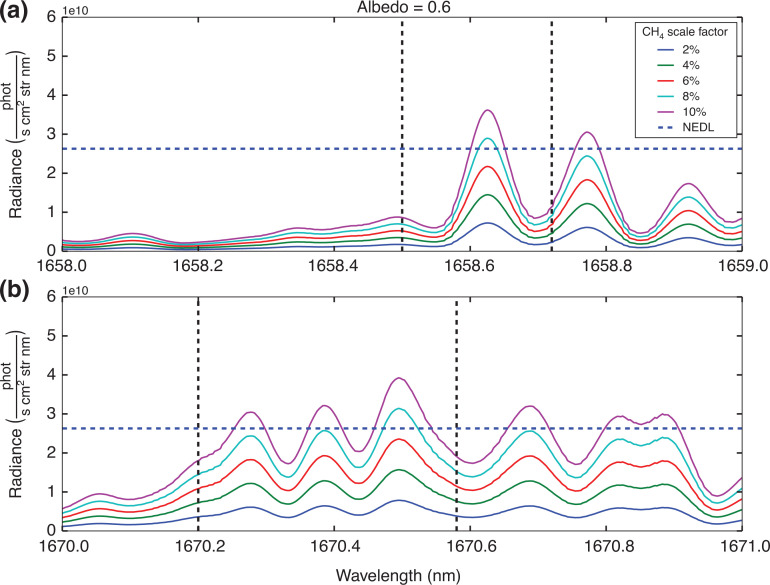
As Fig. 7, with surface albedo increased to 0.6.

Based on the simulation conditions specified in Table 2, consideration is given as to whether or not the individual peaks highlighted in Fig. 4 will exceed the NEDL. Figures 7 and 8 show example results for two different surface albedos, for all the proposed methane concentration levels.

The results in Figs 7 and 8 suggest that detecting changes in concentration of ^13^CH_4_ using individual peaks is unlikely to succeed, with only the highest methane concentrations at the highest albedo levels giving a positive detection and all other residual radiance calculations falling below the NEDL line. However, if we assume the GOSAT sampling pattern which takes three concurrent measurements of the same area [54], by applying Equation 5, and using the mean of ^13^CH_4_ residual radiance peaks, the NEDL is reduced by √3. These are summarised in Table 3.

**Table 3.** Spectral average F_d_ (Equation 5) values summarised for the standard conditions expressed in Table 2. The F_d_ values are shown for each CH_4_ scale given the range of reflectances indicated in Table 1.

CH_4_ total column scale factor	F_d_ Albedo = 0.1 (×10^−10^)	F_d_ Albedo = 0.3 (×10^−10^)	F_d_ Albedo = 0.4 (×10^−10^)	F_d_ Albedo = 0.5 (×10^−10^)	F_d_ Albedo = 0.6 (×10^−10^)
1658.6–1658.65 nm					
×1.02	−1.41	−1.19	−1.08	−0.975	−0.864
×1.04	−1.30	−0.87	−0.652	−0.432	−0.211
×1.06	−1.19	−0.546	−0.219	0.110	0.444
×1.08	−1.08	−0.223	0.213	0.652	1.10
×1.10	−0.974	0.0999	0.644	1.19	1.75
1670.35–1670.55 nm					
×1.02	−1.42	−1.23	−1.13	−1.03	−0.931
×1.04	−1.32	−0.938	−0.742	−0.545	−0.346
×1.06	−1.23	−0.648	−0.355	−0.0582	0.241
×1.08	−1.13	−0.358	0.0327	0.428	0.827
×1.10	−1.03	−0.0688	0.420	0.913	1.41

Considering the results outlined in Table 3 it is clear that the feasibility of detecting any change in ^13^CH_4_ concentration above the NEDL is going to be difficult. The results indicate that the minimum requirements for measuring ^13^CH_4_ concentration with any certainty are a methane source of at least 10% higher concentration than background total column value, with a high surface albedo of 0.3. Although such a combination of conditions is possible, it would likely be limited to wildfire regions such as [48]. Note that the detection factors between the two regions of interest are very similar.

**Figure 9:**
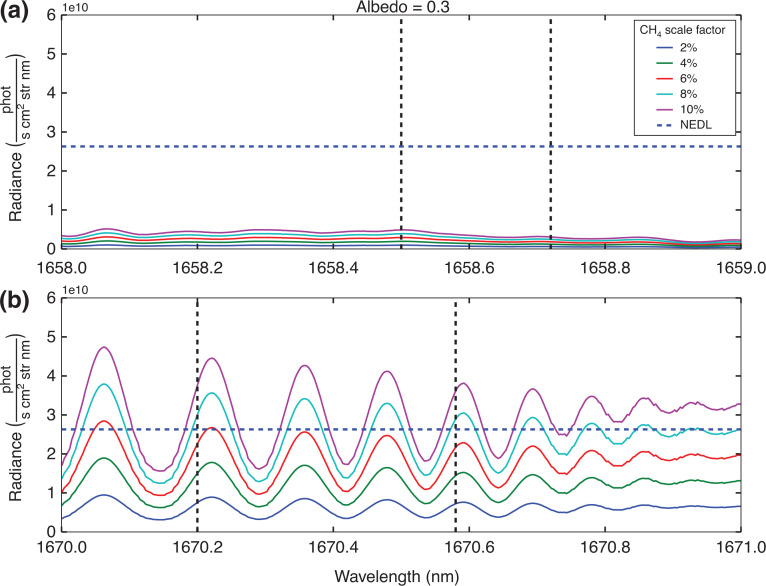
As Figs 7 and 8, with surface albedo increased to 0.3, assuming the standard δ^13^C value is −70‱ as opposed to 0‱.

We note in the section GOSAT and measuring radiance that HITRAN2016 includes an intensity adjustment for methane isotopologues that accounts for natural atmospheric abundance. We now investigate if the detection factors indicated in Table 3 change, if we assume the standard δ^13^C value is −70‱ as opposed to 0‱. To achieve this, we modified the isotopologues intensity in HITRAN2016, by assuming Vienna Pee Dee Belemnite is 0.0010326 as opposed to 0.0011031. Then we reran the scenarios shown in Table 2; the results for the albedo = 0.3 case are shown in Fig. 9.

Figure 9 is interesting because it shows that the ^13^CH_4_ peak at 1658.6 nm is highly sensitive to changes in the assumed δ^13^C value, to the point where changes of the methane column concentration has practically no impact on the residual radiance. While the spectral lines at 1670.4 is not as sensitive to the change in δ^13^C value, and as indicated in Table 4, actually shows an increase in the magnitude of the detection factors.

**Table 4.** Spectral average F_d_ (Equation 5) values summarised for the standard conditions expressed in Table 2, assuming a global standard δ^13^C value of −70‱. The F_d_ values are shown for each CH_4_ scale given the range of reflectances indicated in Table 2.

CH_4_ total column scale factor	F_d_ Albedo = 0.1 (×10^−10^)	F_d_ Albedo = 0.3 (×10^−10^)	F_d_ Albedo = 0.4 (×10^−10^)	F_d_ Albedo = 0.5 (×10^−10^)	F_d_ Albedo = 0.6 (×10^−10^)
1658.6–1658.65 nm					
×1.02	−1.51	−1.49	−1.47	−1.46	−1.45
×1.04	−1.50	−1.45	−1.43	−1.41	−1.39
×1.06	−1.48	−1.42	−1.39	−1.35	−1.32
×1.08	−1.47	−1.39	−1/34	−1.30	−1.25
×1.10	−1.46	−1.36	−1.30	−1.25	−1.19
1670.35–1670.55 nm					
×1.02	−1.42	−1.22	−1.12	−1.03	−0.924
×1.04	−1.32	−0.93	−0.733	−0.535	−0.330
×1.06	−1.22	−0.636	−0.341	−0.043	0.263
×1.08	−1.12	−0.343	0.0512	0.448	0.857
×1.10	−1.02	−0.0494	0.443	0.939	1.450

The HITRAN2016 database suggests that the ^13^CH_4_ spectral lines in the 1670.2–1670.6 nm are made up of a number of different transitions, which exhibit a range of lower state energy values. A number of which are of similar magnitude to those for the main methane isotopologue ^12^CH_4_. While the lower state energy levels for ^12^CH_4_ are significantly larger than those for the ^13^CH_4_ lines in the 1658–1659 nm range, which explains this difference in reactions to changes in the standard δ^13^C values.

In addition to the simulations for the δ^13^C values of 0‱ and −70‱, we also performed an analysis for δ^13^C values of −35‱. Based on the detection factors for the range of δ^13^C value shown in this study, we can plot these variables and determine the conditions where GOSAT can detect differences in δ^13^C values.

Based on the detection values indicated in Tables 3 and 4, and given similar results from an analysis of δ^13^C values of −35‱. We can plot a relationship between the detection values and the surface albedo for a given δ^13^C value.

**Figure 10:**
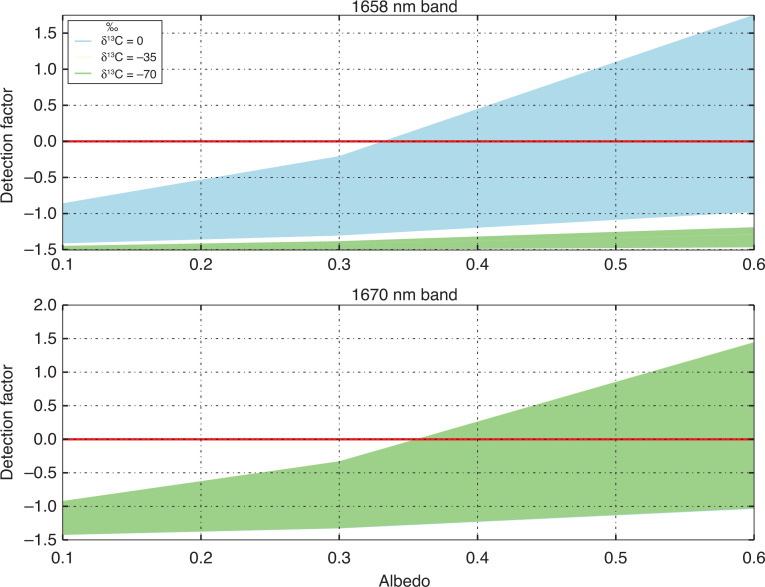
Plot indicating the surface conditions required to generate detection factors >0, thus suggesting a positive detection of δ^13^C. The top panel shows results for the 1658 nm band, and the bottom panel shows results for the 1670 nm band. The light blue area indicates results assuming a δ^13^C natural value of 0‱, light yellow −35‱ and light green −70‱, the solid red line indicates where the detection factor is zero.

Figure 10 is interesting as it shows that the 1658 nm band has more sensitivity to changes in surface reflectance, and total column methane concentration than the 1670 nm band. But only in the case where δ^13^C is assumed to be equal to zero. For the other δ^13^C cases shown in Fig. 10, there are no examples where the detection factor is greater than 0. For the 1670 nm band, although the detection factors are lower in magnitude, the sensitivity to changes in the δ^13^C are minor. These results imply (focusing on the 1670 nm band), that given a significant enhancement in the total methane column, and a high enough surface reflectance, it may be possible to detect changes in the δ^13^C of the measurement. As the detection factor can be related back to a total methane column value, δ^13^C values could be directly estimated. Assuming some knowledge of ^12^CH_4_. Figure 10 suggests that the lowest possible surface albedo of 0.35, requires an enormous methane enhancement of 8% in order to achieve a detection of ^!3^CH_4_, while the highest surface albedo of 0.6 requires an enhancement of 5% or 6%.

The required surface conditions to achieve the above values are not common. Using the aforementioned ADAM dataset (http://adam.noveltis.com/), we can indicate how much of the Earth’s land surface has surface albedo values of at least 0.3. The database suggests that a significant proportion of the Earth has >0.3 surface albedo. Significantly the biomass burning regions indicated in [48] have the required surface albedo, thus suggesting that in the scenarios observed in [48] it would be possible to detect ^13^CH_4_ signals with GOSAT using the methods described in this paper.

### ^13^CH_4_ detectability under high water vapour conditions

Using Equation 6 we can interpret the potential effects of varying water vapour concentration on the spectral averaging factor, given the high water vapour concentration conditions specified in Table 3. Based on the sensitivity factors indicated in Table 5, it is clear that both of the spectral bands we investigate in this paper are affected by the increase in loading of water vapour to some degree. The 1658 nm band is affected to a far less extent than the 1670 nm band (~10%). Most likely because the 1658 nm band is narrower than the 1670 nm band. For both bands the scaling of the methane column has a negligible effect, meaning that the high methane scenarios required to detect ^13^CH_4_ will not be subject to water vapour errors, any more than high surface albedo scenarios. The loading of the water vapour column by 100% is not an unreasonable scenario when considering the difference between mid-latitude scenes and tropical scenes.

**Table 5.** Sensitivity factor for the 1658 nm and 1670 nm wavebands, assuming the low and high water vapour conditions, and a surface albedo of 0.3 specified in Table 2.

Waveband/methane scale	Scale = 2%	Scale = 4%	Scale = 6%	Scale = 8%	Scale = 10%
1658.6 nm S_f_	1.00121	1.00126	1.00127	1.00125	1.00125
1670.35 nm S_f_	1.0113	1.00113	1.0113	1.0112	1.0112

### Comparisons with GOSAT-TANSO-FTS L1B data

**Figure 11:**
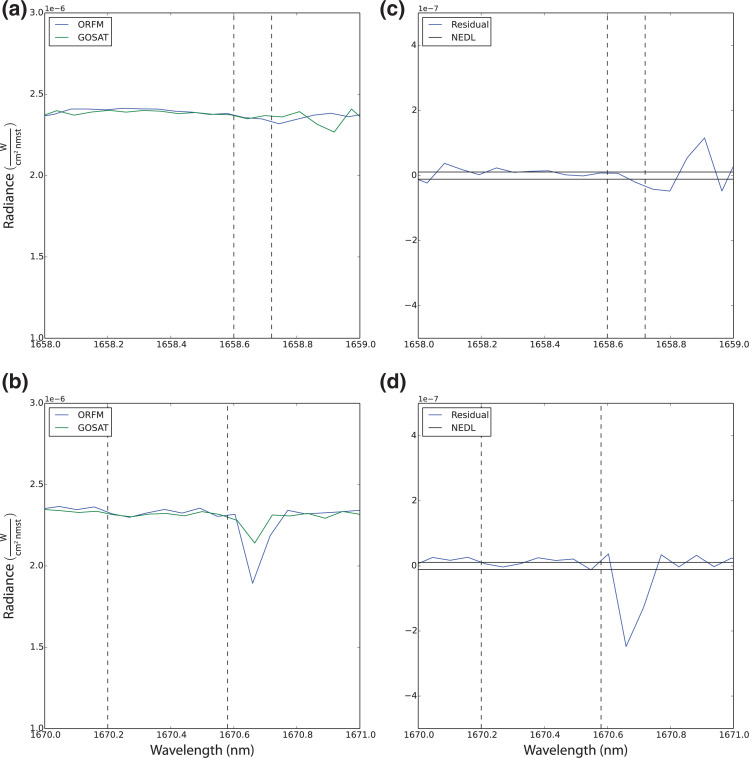
Comparison of simulated spectrum against GOSAT L1B data. In this particular example, the surface reflectance is 0.33, solar zenith angle is 46° and satellite view zenith angle is 24°, the L1B data was captured at a high latitude on 01/06/2016 at 61.7°N, 173°E: (a) highlights the 1658–1659 nm spectral region, where the two dashed lines highlight the exact region of ^13^CH_4_ activity. The blue lines shows simulated ORFM data and the green line shows GOSAT L1B data; (b) highlights the 1670–1671 nm spectral region, where the two dashed lines highlight the exact region of ^13^CH_4_ activity. The blue lines show simulated data and the green line shows GOSAT L1B data; (c) shows the residual radiance between the simulated spectrum and the L1B data in the 1658–1659 nm range, the horizontal solid lines represent the FTS NEDL and the two dashed lines highlight the exact region of ^13^CH_4_ activity; (d) shows the residual radiance between the ORFM spectra and the L1B data in the 1670–1671 nm range, the horizontal solid lines represent the FTS NEDL and the two dashed lines highlight the exact region of ^13^CH_4_ activity.

The assessments outlined above are predominately based on using synthetic data; it is therefore important to determine if variations in ^13^CH_4_ can occur in real measured GOSAT-TANSO-FTS L1B spectra. Based on the method described in the section Applying to GOSAT-TANSO-FTS L1B spectra, direct comparisons can be made between the synthetic data and L1B data. Figure 11 represents an interesting counter perspective to the results shown in the previous sections. Figure 11(a) shows largely good agreement between the ORFM and L1B spectra, aside from two spectral line absorption points in the L1B data, not present in the ORFM simulation [highlighted in Fig. 11(b)], possibly due to residuals not captured in the ORFM simulations. Despite this, there is very little difference between the radiances of the ORFM and L1B spectra in the highlighted portion.

However, considering Fig. 11(c), the closely matching spectra indicate a reasonable fit from the method discussed in the section ‘Applying to GOSAT-TANSO-FTS L1B Spectra’. Focusing on the highlighted region in Fig. 11(c) and (d) show a variation in the radiance of the ^13^CH_4_ region, suggesting potential for GOSAT measuring changes in ^13^CH_4_ over background concentrations. An important point to consider is the water vapour spectral line peak at 1670.68 nm; the ORFM simulations suggest a higher concentration of water vapour in the atmosphere based on the depth of the spectral line than the L1B data. The section ‘^13^CH_4_ Detectability under High Water Vapour Conditions’ shows that the methane isotopologue spectral lines are heavily influenced by water vapour; therefore the lower concentrations of water vapour in the L1B spectrum, combined with the high reflectance value may allow for variation in the ^13^CH_4_ concentration to become more obvious than suggested in the simulation studies. Therefore, in some scenarios, a high surface reflectance of at least 0.6 may not be necessary.

## Discussion

The range of scenarios where ^13^CH_4_ can be detected is very small. We acknowledge that this method is not as sophisticated or as accurate as a full sensitivity analysis using Rodgers’ optimal estimation method. However, we argue that the benefits of the method shown in this study is its simplicity, such that a quick analysis can be performed by a lay person interested in the subject area, or it could be used to teach advanced school students, or early year university students. Indeed, scientists interested in quickly determining the sensitivity of a trace gas species could use this method as a quick first step, before committing to further analysis. The most complex part of this study is the RTM, and here we use two well-established RTMs to achieve the goals of this study. RTM development is a far more complex task than developing a retrieval algorithm, and independently developing an RTM would no longer make this study simple or quick. There are significantly more open source RTMs available than retrieval algorithms, this variety in RTMs mean that there should be sufficient ranges in solutions and methods that allow for characterisation of any errors in the forward models.

The detection analysis outlined in the section Detecting changes in ^13^CH_4_ signal is based on the total column of methane detection of δ^13^C, this method is potentially limiting to a degree since this does not take into account KIEs in the upper troposphere and lower stratosphere due to the destruction of methane. However, as ^13^CH_4_ concentration is low, and the KIE factors are less than those at the surface, such factors are unlikely to have a significant impact on the results. In addition, atmospheric air currents interfere with the total column and thus will dampen the signal of δ^13^C in the total column, as opposed to *in situ* measurements. There are currently no studies that investigate this effect, but we can assume that the δ^13^C differences between source types will be even smaller.

Other error sources include the spectroscopy and the forward model. The HITRAN2016 database in combination with the SCIATRAN forward model assumes a Voigt profile for all methane lines in the GOSAT spectral sensitivity ranges. The Voigt profile has been generally assumed for methane spectral regions in the past, however this shape is now acknowledged to be no longer sufficient [41]. The current HITRAN2016 database does not include the parameters necessary to estimate non-Voigt line shapes for methane; however, it is anticipated that future updates will include these. We therefore accept that there will be spectroscopic errors present in this study. Following on from the HITRAN database, the next largest error sources are likely to arise from SCIATRAN, generated from inaccuracies in recreating the absorption or radiance spectra from a given set of atmospheric inputs.

The metrics F_d_ and S_f_ give a useful indication of the feasibility of detecting ^13^CH_4_, and can be used to further inform a user about the feasibility of detection over a wider variety of atmospheric and surface conditions than shown in this study. However, caution must be applied since, as highlighted in Table 5, the influence of water vapour on the ^13^CH_4_ peaks might well lead to false positive values of F_d_, and therefore create an incorrect inference of isotopologues detection.

Although we briefly looked at methane isotopologues absorption in the GOSAT TIR band, we did not investigate this in depth. This is despite the fact that the isotopologues indicated much larger optical depth than their equivalent in the SWIR. However, there is significant evidence to suggest that the spectroscopy of methane in the TIR is not nearly as advanced as that in the SWIR [62], which is important given the short wavebands used in this study. In addition to the high levels of background interference on the ^13^CH_4_ spectral lines observed in Fig. 6.

An obvious next or alternative step would be to perform retrievals of the methane isotopologues using the Total Column Carbon Observing Network (TCCON, [63]). TCCON relies on solar occultation measurements as opposed to solar backscatter, and operates at a much higher SNR and spectral resolution than GOSAT. The key disadvantage to TCCON is that it is limited to a small number of sites all over the globe, and cannot be as beneficial to global studies as satellites such as GOSAT. This work has been shown in a separate study, indicating that even with the improved SNR of the TCCON instruments, there are still significant challenges with retrievals of methane isotopologues [64].

## Conclusions

In this paper we investigated the potential to detect the second most common methane isotopologue (^13^CH_4_) using the GOSAT-TANSO-FTS instrument. The ratio of the main methane isotopologues has been shown to be able to differentiate between different methane source types, and could be a useful tool in linking global bottom-up emissions with top-down emissions.

We use a simple and quick residual radiance method in order to investigate the benefit of such techniques, in the wider context of the more sophisticated methods based on Rodgers’ optimal estimation techniques. We argue that the residual radiance technique is useful as a simple and quick method for analysing spectral regions for sensitivity to specific trace gases.

The results of this study generally suggest that detecting the second most important methane isotopologue is difficult in most circumstances, apart from unique circumstances such as large biomass burning events. Using these techniques we find that detections of ^13^CH_4_ with GOSAT can only occur with surface albedos of >0.3, assuming at least an 8% enhancement in the methane total column. This total column requirement is reduced with increasing surface albedo. In the context of a world where El Nino events are likely to become more frequent, it is possible that the required conditions for ^13^CH_4_ detection using this technique, may become more common.

We perform the assessment using the general assumption of δ^13^C = 0 globally as this is built into the HITRAN databases. However, we also investigate the effects of detecting the ^13^CH_4_ isotopologue using different values of δ^13^C, ranging up to −70‱. We find that the spectral lines in the 1670 nm waveband are unaffected by the change in δ^13^C, while other spectral regions are significantly affected by this change.

We also assess the suitability of the TIR region for methane isotopologues, and find that although the optical depth of ^13^CH_4_ is greater than that in the SWIR region, the dominance of background trace gases, and the unknowns in the spectroscopy of the region make this region less attractive than the SWIR.

## Glossary

Table 6 provides a table of key terms and acronyms.

**Table 6.** Glossary of key terms and acronyms used in this paper.

Term	Details
ADAM	A surface reflectance Database for ESA’s Earth observation Missions; database of surface reflectance
CTM	Chemistry transport model; mathematical model simulating the transport of trace gases in the atmosphere
δ^13^C	Ratio of ^13^CH_4_ to ^12^CH_4_ compared to the Vienna Pee Dee Belemnite standard
ESA	European Space Agency
FTS	Fourier transform spectrometer; sensor on GOSAT designed to measure trace gases
GHG	Greenhouse gas(es)
GOSAT	Greenhouse Gases Observing Satellite; Satellite launched in 2009
HITRAN	High resolution transmission; database of spectroscopic parameters
L1B	Level 1B data; first stage processed data from the instrument, representing the spectral response
IC	Information content; mathematical technique to assess quality of trace gas retrievals
Isotopologue	Molecule with at least one atom containing non-periodic table number of neutrons
JAXA	Japanese Aerospace Exploration Agency
KIE	Kinetic isotope effects; determines the rate of reactions based on isotopic make up of molecule
MIPAS	Michelson Interferometer for Passive Atmospheric Sounding; instrument on ENVISAT
NEDL	Noise equivalent radiance; instrument noise represented as radiance values
NIES	National Institute for Environmental Studies
ORFM	Oxford Reference Forward Model; radiative transfer model developed at the University of Oxford
RTM	Radiative transfer model; model designed to simulate radiation transfer through a medium
SCIAMACHY	SCanning Imaging Absorption SpectroMeter for Atmospheric CHartographY; instrument on ENVISAT
SCIATRAN	Name of radiative transfer model developed at the University of Bremen
SNR	Signal to noise ratio
SWIR	Shortwave infrared; portion of the electromagnetic spectrum ~1–3 μm
TANSO	Thermal and near Infrared Sensor for Carbon Observations; instrument onboard GOSAT
TCCON	Total Column Carbon Observing Network; series of upwards viewing FTSs located around the world
TIR	Thermal infrared; portion of the electromagnetic spectrum ~>5 μm to microwave
TROPOMI	Tropospheric Monitoring Instrument; instrument based on Sentinel-5P

## Data Availability

HITRAN2016 data is available from https://hitran.org/ The OFRM is available through the website http://eodg.atm.ox.ac.uk/RFM/ SCIATRAN is available through http://www.iup.uni-bremen.de/sciatran/index.html The GOSAT L1B data is available through the GOSAT Data Archive Service https://data2.gosat.nies.go.jp/index_en.html The ORFM and SCIATRAN simulations used in this article are fully reproducible given the input parameters provided in this article.
